# 小细胞肺癌合并腺癌MDT治疗报道及文献综述

**DOI:** 10.3779/j.issn.1009-3419.2021.102.37

**Published:** 2021-11-20

**Authors:** 子涵 瞿, 洁薇 刘, 锋 罗, 潞 李, 玲玲 朱, 清华 周

**Affiliations:** 610041 成都，四川大学华西医院肺癌中心；四川省肺癌研究所 Lung Cancer Center, West China Hospital, Sichuan University; Sichuan Lung Cancer Institute, Chengdu 610041, China

**Keywords:** 肺肿瘤, 混合型小细胞肺癌, 敏感性基因突变, 组织活检, 基因检测, Lung neoplasms, Mixed small cell lung cancer, Sensitive gene mutations, Biopsy, Gene testing

## Abstract

小细胞肺癌（small cell lung cancer, SCLC）是肺癌中恶性程度和死亡率最高的类型。目前的一线标准治疗方式仍是以依托泊苷和铂类为主的化疗方案。然而，对于一线治疗后进展的SCLC，治疗方式仍非常有限。由于目前对SCLC一线耐药的分子机制尚未明了，而且一线耐药后的精准医疗策略仍处于临床前期阶段，因而在SCLC一线治疗进展后，进行二次活检及基因检测的比例很低。本文通过报道1例初次诊断为SCLC的中年女性，病程中多次活检发现肿瘤组织病理学类型出现具有敏感基因突变的腺癌以及小细胞癌的反复转变，提示其可能为SCLC中的特殊亚型——混合型小细胞肺癌（mixed small cell lung cancer, M-SCLC）。本例患者在治疗过程中先后使用了放化疗、免疫治疗及靶向治疗，目前生存时间已达2年8个月。本文通过病例报道及回顾性文献复习，旨在探讨对一部分可能一开始就呈现混合型病理组织类型或在治疗后出现组织类型改变的SCLC，疾病进展时有必要再次活检明确病理组织学类型及基因学检测，寻找潜在治疗靶点，以便给予基于分子标志物检测结果的精准治疗，为患者提供尽可能长的生存获益。

## Abstract (en)

Small cell lung cancer (SCLC) is the most malignant lung cancer with the highest mortality. At present, the first-line standard treatment is still based on Etoposide and Platinum chemotherapy. However, for SCLC that progresses after first-line therapy, the treatment options are still very limited. Since the molecular mechanism of first-line drug resistance of SCLC is still unclear, and the precision medicine strategy after first-line drug resistance is still in the pre-clinical stage. The proportion of secondary biopsy and genetic testing is very low after the progress of first-line treatment of SCLC. In this study, we report a case of a middle-aged woman who was first diagnosed with SCLC. Adenocarcinoma with sensitive gene mutations and repeated changes of small cell carcinoma were detected by multiple biopsies during the course of the disease, suggesting that the patient may be a special subtype of SCLC - mixed SCLC (M-SCLC). In this case, the patient has been treated with radiotherapy and chemotherapy, immunotherapy and targeted therapy successively, and the survival time has reached 2 years and 8 months. Through the case report and literature review retrospectively, this study aimed to explore the part patients may start to present hybrid histopathologic types or tissue type change after treatment of SCLC. Biopsy pathologic histology and genetic testing is necessary after disease progression to look for potential therapeutic targets, so as to give precise treatment based on molecular markers detection results and provide the patient with the benefit of survival for as long as possible.

## 1 病例介绍

患者，女性，56岁，因左侧胸背部剧烈疼痛伴咯血1^+^年就诊。既往无烟酒史，无传染病史，有肿瘤家族史。胸部计算机断层扫描（computed tomography, CT）提示：左肺上叶占位伴肺门及纵隔淋巴结增大。纤维支气管镜提示：左上叶上支开口浸润性新生物阻塞。病理诊断（Q1829316，2018年8月20日）示：（左肺上叶）小细胞癌。免疫组化示：磷酸烯醇丙酮酸羧激酶（phosphoenolpyruvate carboxykinase, PCK）和上皮膜抗原（epithelial membrane antigen, EMA）均（点状+）、P40（-）、甲状腺转录因子-1（thyroid transcription factor-1, TTF-1）（+）、嗜铬粒蛋白A（chromogranin A, CGA）（-）、CD56（+）、突触融合蛋白（synataxin, Syna）（部分+）、细胞角蛋白（cytokeratin 7, CK7）（-）、Ki-67（+, 70%）、程序性死亡配体1（programmed cell death ligand 1, PD-L1）（-）。全身骨扫描示：左侧髂骨骨代谢增高灶，肿瘤骨转移可能。

随后患者接受6个周期EP（依托泊苷+顺铂）方案化疗，胸部病灶放疗原发肿瘤计划肿瘤靶区（planned gross target volume, PGTV）（左上肺原发灶+纵隔转移淋巴结+高危淋巴结引流区），调强适形放疗（intensity modulated radiation therapy, IMRT）60 Gy/30 F/6 wk；髂骨转移灶放疗PGTV（左髂骨翼转移灶），IMRT 60 Gy/20 F/4 wk。综合疗效评价为疾病缓解（partial response, PR）。患者拒绝行预防性颅脑照射（preventive craniocerebral irradiation, PCI）治疗。

放化疗结束后3个月患者因突发视物旋转伴明显恶心、呕吐，行急诊头颅CT提示颅内多发转移瘤。立即予以甘露醇脱水治疗及头部放疗PGTV（脑转移灶），三维适形放疗（three dimension conformal radiotherapy, 3D-CRT）42 Gy/14 F/3 wk。放疗后颅内病灶疗效评价为PR。腹部CT提示双侧肾上腺多发转移瘤。考虑患者疾病进展（progressive disease, PD），遂开始行二线TC（脂质体紫杉醇+卡铂）方案化疗联合重组人血管内皮抑制素治疗。

二线治疗2个周期后复查胸腹部CT提示：左侧中量胸腔积液，双侧肾上腺多发转移瘤较前明显增大。行左侧胸腔穿刺置管引流术，胸水送脱落细胞学显示（胸水涂片及细胞块）：查见腺癌细胞。免疫细胞化学染色示：TTF-1（+）、Napsin-A（+）、CD56（-）、Syn（-）、CgA（-），倾向肺来源。肿瘤细胞间变性淋巴瘤激酶（anaplastic lymphoma kinase, ALK）（-）、c-ros肉瘤致癌因子-受体酪氨酸激酶（ROS proto-oncogene 1, receptor tyrosine kinase, ROS1）（-）。随即接受三线单药帕博利珠单抗（200 mg, *ivgtt*, *q3w*）免疫治疗共2个周期及双侧肾上腺放疗PGTV（双侧肾上腺转移灶），容积旋转调强技术（volume modulated arc therapy, VMAT）50 Gy/25 F/5 wk。2个周期后综合疗效评价为疾病稳定（stable disease, SD）（左侧肺门占位SD，双侧肾上腺转移瘤PR）。患者外周血基因检测[突变阻滞扩增系统聚合酶链反应（amplification refractory mutation system polymerase chain reaction, AMRS-PCR）]结果示：表皮生长因子受体（epidermal growth factor receptor, *EGFR*）L858R（+, 36.34%）。遂行吉非替尼250 mg *qd*治疗，靶向治疗后1个月复查胸部CT提示：左肺门病灶稍增大，纵隔淋巴结较前增多、增大。肿瘤再次进展，随后换用依托泊苷单药化疗联合安罗替尼抗血管生成治疗。治疗1个月后复查胸腹部CT后综合疗效评价为SD（左侧肺门占位稍增大）。服药期间患者出现手足综合症，症状明显，自行停药。

停药1个月后患者无明显诱因出现无痛性血尿，为明确诊断行CT尿路造影提示：膀胱左侧局灶性增厚，强化，肿瘤性病变？考虑膀胱恶性肿瘤可能性大。排除手术禁忌，于2020年11月20日在全麻下行“经尿道膀胱肿瘤电切术”，术后病理诊断：（膀胱）恶性肿瘤，免疫组化染色：肿瘤细胞呈PCK（点灶状+）、Syn（+）、CgA（+）、CD56（+）、TTF-1（+）、GATA-3（-）、CDX2（-）、Ki-67（MIB-1）阳性率约为80%，结合病史支持为肺小细胞癌转移。术后患者恢复可，持续随访中。患者诊疗过程见图 1。

**图 1:**
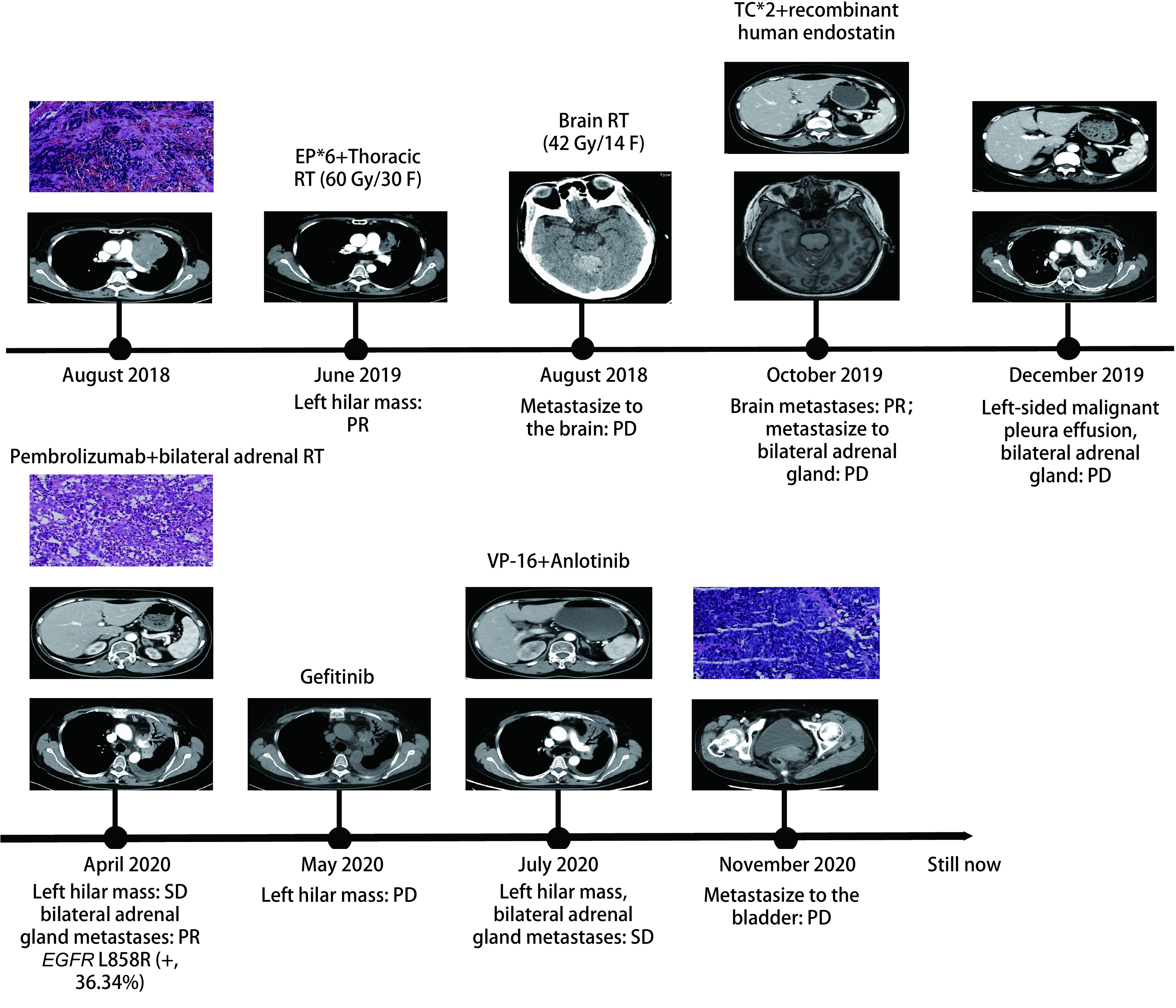
患者诊疗经过概述 Overview of the patient's diagnosis and treatment. ED: extensive disease; RT: radiation therapy; EP: etoposide+cisplatin; TC: paclitaxel+carboplatin; PR: partial response; PD: progressive disease; SD: stable disease; VP-16: etoposide; EGFR: epidermal growth factor receptor.

## 2 文献回顾及讨论

本文报道1例首诊为SCLC，接受标准一、二线治疗后出现PD，多次病理学活检提示肿瘤组织出现携带敏感性基因突变的腺癌与SCLC组织类型转变的患者，并且于PubMed上检索既往报道SCLC合并腺癌成分的类似病例，对患者的临床特点（性别、年龄、肺癌组织学类型、肿瘤分期）、合并基因突变、接受治疗/疗效评价及总生存期进行回顾及归纳（表 1）。

**表 1:** 文献回顾SCLC合并腺癌患者临床特征文献回顾 Literature review of clinical features of the patients with SCLC complicated with adenocarcinoma

Reference	Age/Gender	Smoking	Pathological diagnosis	Diagnosis specimen type	Mutation gene	Stage	Treatment	Response	OS (mon)
Tatematsu *et al*^[26]^	69/M	Yes	SCLC/ADC	Biopsy specimen	*EGFR* L858R	Ia	Surgery and adjuvant chemotherapy	-	-
Tatematsu *et al*^[26]^	65/M	Yes	SCLC/ADC	Resected tumor (cytological diagnosis of SCLC prior to surgery)	*EGFR* Ex19del	Ia	Adjuvant chemotherapy and radiotherapy	-	-
Tatematsu *et al*^[26]^	36/F	No	SCLC/ADC	Resected tumor (diagnosis of ADC with a biopsy prior to surgery)	*EGFR* L858R	IV	Surgery	-	-
Siegele *et al*^[27]^	82/M	Yes	SCLC/ADC	Resected tumor	*EGFR* D855H	Ia	Surgery and adjuvant chemotherapy	-	Lost to follow up
Shi *et al*^[28]^	71/M	Yes	SCLC/ADC	Resected tumor	*EGFR* L858R	I†	Surgery	-	12
Lu *et al*^[29]^	62/F	No	SCLC/ADC	Resected tumor	*EGFR* Ex19del	IIIa	Surgery	-	-
Wakuda *et al*^[30]^	73/M	Yes	SCLC/ADC	Resected tumor	*EGFR* G719A	IIb	Surgery	-	-
Iijima *et al*^[31]^	63/M	Yes	SCLC/ADC	Resected tumor	*EGFR* Ex19del	IIb	Surgery	-	-
Iijima *et al*^[31]^	76/M	Yes	SCLC/ADC	Resected tumor	*EGFR* Ex19del	IIIa	Surgery	-	-
Lu *et al*^[20]^	62/F	No	SCLC/ADC	Resected tumor	*EGFR* Ex19del	IIIa	Surgery and adjuvant chemotherapy	-	22
Norkowski *et al*^[32]^	66/F	Yes	SCLC/ADC	Resected tumor	*EGFR* Ex19del	IIIa	-	-	120
Norkowski *et al*^[32]^	62/M	No	SCLC/ADC	Resected tumor	*EGFR* Exon 18 G719A, Exon 21 L833_V834delinsFL	IIIa	-	-	8
Norkowski *et al*^[32]^	45/F	Yes	SCLC/ADC	Resected tumor	Exon 21 L858R	IIIa	-	-	12
Ye Guo *et al*^[22]^	61/M	Yes	SCLC/ADC	Biopsy specimen	*EGFR* Ex19del	ED	Chemotherapy and Erlotinib	SD	-
Fukui *et al*^[33]^	62/F	No	SCLC/ADC	Resected tumor	*EGFR* L858R	IIIb	Surgery	-	-
Lin *et al*^[10]^	66/F	No	SCLC/ADC	Resected tumor	*EGFR* L858R	IIIa	-	-	-
Lin *et al*^[10]^	77/F	No	SCLC/ADC	Resected tumor	*EGFR* L858R	IIIa	Erlotinib	NS	-
Lin *et al*^[10]^	63/F	No	SCLC/ADC	Resected tumor	*EGFR* G719A	IVb	Afatinib	NS	-
Takagi *et al*^[34]^	70/F	No	SCLC/ADC	Biopsy specimen	*EGFR* L861Q	IVb	Chemotherapy	PR	-
Tanaka *et al*^[23]^	67/M	No	SCLC/ADC	Biopsy specimen	*EGFR* Ex19del	IVb	Chemotherapy and Afatinib	PR	-
Toyokawa *et al*^[15]^	72/M	Yes	SCLC/ADC	Resected tumor	*EGFR* Ex19del, *EML4*-*ALK*	Ib	Surgery	-	-
^†^: staging details not specific. ADC: adenocarcinoma; SCLC: small cell lung cancer; ED: extensive disease; OS: overall survival; EML4-ALK: echinoderm microtubule-associated protein-like 4-anaplastic lymphoma kinase; F: female; M: male.									

由于一部分肺腺癌患者接受EGFR酪氨酸激酶抑制剂（tyrosine kinase inhibitors, TKIs）治疗后以出现SCLC病理学改变作为获得性耐药机制，所以本文排除了接受病理活检前接受EGFR-TKIs治疗的患者。21例混合型小细胞肺癌（mixed small cell lung cancer, M-SCLC）合并*EGFR*敏感性基因突变的患者纳入文献回顾，患者中位年龄65.24岁（范围：36岁-82岁），其中女性11例（11/21, 52.38%），男性10例（10/21, 47.62%），52.38%（*n*=11）有明确吸烟史。80.95%（*n*=17）的患者通过手术切除标本获得病理诊断，其中1例患者术前经穿刺活检诊断为单纯型SCLC，另外1例诊断为肺腺癌。肿瘤分期方面仅有5例患者为Ia期-Ib期，其余16例患者于就诊时已处于（局部）晚期。关于基因型分析，42.86%（*n*=9）的患者合并*EGFR*外显子缺失突变（19del），33.33%（*n*=7）的患者合并*EGFR*外显子21突变，14.29%（*n*=3）的患者合并*EGFR*外显子18 G719A突变，另外，各有1例（4.76%）患者分别携带*EFGR* L833_V834delinsFL和*EGFR* D855H罕见突变。尽管本研究纳入的均为携带肿瘤驱动基因的患者，但仅有3例患者接受EGFR-TKIs治疗，大部分患者仍采用手术、全身化疗或局部放疗的治疗方法。由于本研究纳入的接受EGFR-TKIs治疗的样本量有限，靶向治疗对M-SCLC是否有效还需要后续大样本的临床研究证实。

M-SCLC为SCLC的特殊病理类型，肿瘤组织中SCLC和任何非小细胞肺癌（non-small cell lung cancer, NSCLC）成分共存，最常见的NSCLC组织学亚型是大细胞癌（large cell carcinoma, LC）或大细胞神经内分泌癌（large cell neuroendocrine carcinoma, LCNEC），其余少见的为腺癌、鳞癌，极少合并梭形细胞癌或巨细胞癌（如果是LC或LCNEC至少应含10%肿瘤组织才能诊断）^[1, 2]^。M-SCLC的发生率为2%-28%^[3]^，该差异与肿瘤组织样本大小、完整性、病理诊断水平相关。由于SCLC大都位于段支气管以上，诊断主要依靠支气管镜、肺泡灌洗液或细针穿刺活检等方式，获得的组织样本有限，因此诊断率较低。而在手术标本中更容易被诊断，可能与出现在周围支气管及肿瘤切除率更高有关^[4]^。

SCLC极易发生远处转移，前期通过对小鼠SCLC模型和循环肿瘤细胞（circulating tumor cells, CTCs）的研究发现，与NSCLC类似淋巴结转移在SCLC中很常见^[5, 6]^，但血行转移是SCLC的主要转移途径，肿瘤通常会转移至胸膜腔和远处脏器^[7]^，这一现象与本文报道的患者出现的情况类似，患者病程中先后出现纵隔淋巴结转移、颅内转移、胸膜转移及膀胱转移。

本例患者随着疾病进展发生“SCLC-腺癌-SCLC”的病理组织类型改变且合并敏感基因突变，该现象或许可以解释为：①肿瘤组织中一开始就合并腺癌成分；②疾病进程中出现第二原发肿瘤；③腺癌成分为原发肿瘤分化而来；④治疗后SCLC发生细胞形态学改变。该患者通过支气管镜活检确诊SCLC，由于获取的样本量有限，未能准确反映该肿瘤的病理学特征，容易误诊；其次，患者除左肺门原发病灶外，其余胸腔内未见异常占位，因此胸腔积液中腺癌细胞来源于原发肿瘤的可能性大。另外，何佳林等^[8]^对175例SCLC患者接受放化疗前的活检组织及死亡后的尸检标本进行细胞组织形态的对比分析发现，治疗前约8.6%的患者为M-SCLC，而在单纯型SCLC中约9.7%的患者于治疗后出现组织学类型的改变，主要发生在原发灶与转移灶之间。Rudin等^[9]^报道13%-45%的单纯型SCLC治疗后出现获得性化疗耐药，其表现为细胞形态改变及混合型组织学类型。

目前，SCLC合并腺癌的发生机制及组织起源仍不明确。*TP53*、*RB1*抑癌基因突变是SCLC发生所必需的分子生物学事件。Lin等^[10]^证实合并腺癌的M-SCLC中两种肿瘤组织中均存在*EGFR*、*TP53*、*RB*等基因突变，提示两者具有共同的组织遗传学背景。Swanton等^[11]^发现腺癌成分和SCLC或许是来源于同一肿瘤干细胞的不同亚克隆突变，且早于*TP53*基因突变。肿瘤干细胞的多个亚克隆受不同肿瘤微环境的作用而发生基因突变（例如*PI3KCA*^[12]^、*Notch*^[13]^等），进而影响肿瘤细胞的分化。另外，大部分SCLC起源于神经内分泌细胞，但有少数SCLC和肺腺癌的组织学起源相同，为II型肺泡上皮细胞^[14]^。Toyokaw等^[15]^报道1例合并腺癌成分且携带*ALK*及*EGFR*基因突变的M-SCLC患者，其仅在SCLC中检测到*ALK*重排，提示*ALK*重排或许与SCLC的发生有关。临床病理特征方面，携带敏感基因突变的患者多为不或少吸烟的女性，表明在分子生物学上类似于传统肺腺癌而非SCLC。

M-SCLC与SCLC临床特点类似，多见于有吸烟史的中年男性，而对于无或有轻度吸烟史的年轻女性SCLC患者，疾病进展时应考虑到M-SCLC的可能，有必要重复活检明确诊断。SCLC多表现为肺门占位及纵隔淋巴结肿大，而合并腺癌成分的则更多见于周围支气管，且部分伴有恶性胸腔积液^[16]^，相反合并鳞癌成分的大多为中央型肺癌^[17]^。因此，通过细针穿刺活检或胸水脱落细胞学检查确诊的周围型SCLC，也应考虑到M-SCLC的可能。

尽管当前诊疗技术已经可以区分SCLC的生物学亚型，但在治疗方面并未根据分型不同而详细划分治疗方案，仍采用手术切除、放化疗、靶向治疗和免疫治疗等多学科诊疗（multi-disciplinary therapy, MDT）手段。初始治疗方法与肿瘤分期相关，在临床实践中推荐使用肿瘤原发灶-淋巴结-转移（tumor-node-metastasis, TNM）分期，能较美国退伍军人肺癌协会（Veterans Administration Lung Study Group, VASLG）分期系统（局限期和广泛期）更为精确地提供原发灶部位、淋巴结及远处转移灶等解剖信息，有助于临床医生确定最优的治疗策略。He等^[18]^回顾了784例M-SCLC患者的临床资料，发现极早期（Ia期-Ib期）患者的主要治疗手段为手术切除和以铂类为基础的辅助化疗，对于IIa期-IV期的患者可同时接受全身化疗联合局部放疗，对已合并远处转移的患者则给予全身化疗联合或不联合免疫治疗。与单纯型SCLC不同的是，晚期M-SCLC化疗敏感性较单纯型SCLC差，预后较差，可能与混杂了NSCLC成分相关^[19]^。

携带肿瘤驱动基因的SCLC非常少见，*EGFR*突变的发生率不到5%，而在M-SCLC中可达15%-20%^[16]^，多见于无或有轻度吸烟史、年轻女性且混合腺癌成分的SCLC^[20]^。已有个别案例^[21-23]^报道小分子TKIs对SCLC合并肺腺癌的患者有效，这表明靶向治疗可能是合并敏感性基因突变的M-SCLC患者的一种新的选择。但TKIs治疗混和腺癌成分且携带敏感基因突变的M-SCLC临床资料有限，其临床疗效仍待后续研究进一步证实。

免疫治疗现已用于SCLC的三线治疗，SCLC存在基因组及染色体高度不稳定性，M-SCLC作为SCLC的特殊类型，理论上其可能对免疫治疗更加敏感。然而，目前尚无免疫检查点抑制剂（immune checkpoint inhibitors, ICIs）治疗的报道，免疫治疗对于M-SCLC的疗效仍需未来进行深入探讨。

本研究报道的病例，在检测到腺癌组织学类型且合并敏感靶点突变时，先后使用了免疫治疗、靶向治疗及抗血管生成治疗。从疗效评价分析，免疫治疗联合放疗期间，肺部病灶为SD，肾上腺病灶达到PR，表明免疫治疗可能有一定疗效。尽管过去有病例报道合并腺癌成分的M-SCLC患者接受靶向治疗后疗效明显，但本研究提及的患者在接受靶向治疗1个月后即出现PD，表明合并敏感靶点突变的SCLC并不一定对EGFR-TKIs靶向治疗敏感。提示M-SCLC可能与*EGFR*基因突变的单纯肺腺癌有不同的疾病起源或分子驱动事件。另外，在靶向治疗进展后，安罗替尼联合依托泊苷单药化疗方案对患者胸部及肾上腺病灶控制稳定，虽然患者不能耐受毒副反应，仍能提示安罗替尼对复发、转移的SCLC有一定的抗肿瘤活性。并且前期临床试验^[24, 25]^已证实在复发难治的SCLC中安罗替尼较安慰剂能明显改善患者的无疾病进展生存期（4.1个月*vs* 0.7个月，*P* < 0.000, 1）、总生存期（7.3个月*vs* 4.9个月，*P*=0.002, 9）并提高疾病控制率（71.6% *vs* 13.2%, *P* < 0.000, 1），且不良反应可控。

对于广泛期患者，选择全身化疗为基础同时联合局部放疗、靶向治疗、免疫治疗等多种治疗手段是主要的治疗模式，需要依据患者病情制定个体化治疗策略。

本研究结果帮助我们对合并肺腺癌成分的M-SCLC有了进一步的认识，在SCLC合并腺癌或不吸烟的患者中，应考虑是否携带*EGFR*突变或*ALK*重排，进行基因检测和重复活检有助于制定个体化MDT治疗策略。最佳的治疗手段应根据治疗过程中每个阶段的优势组织学类型来决定。明确肿瘤的组织学形态后，全基因组下一代测序技术（next generation sequencing, NGS）分析可能会揭示决定这些肿瘤的组织学和临床特征的因素。在重复活检有困难时，肿瘤标志物、循环肿瘤细胞以及利用血浆样本进行敏感基因的突变检测，可能会对医生和患者下一步治疗的决策有重要的帮助。
